# Acceptance towards Monkeypox Vaccination: A Systematic Review and Meta-Analysis

**DOI:** 10.3390/pathogens11111248

**Published:** 2022-10-28

**Authors:** Juan R. Ulloque-Badaracco, Esteban A. Alarcón-Braga, Enrique A. Hernandez-Bustamante, Ali Al-kassab-Córdova, Vicente A. Benites-Zapata, D. Katterine Bonilla-Aldana, Alfonso J. Rodriguez-Morales

**Affiliations:** 1 Escuela de Medicina, Universidad Peruana de Ciencias Aplicadas, Lima 15023, Peru; 2 Sociedad Científica de Estudiantes de Medicina, Universidad Peruana de Ciencias Aplicadas, Lima 15023, Peru; 3 Sociedad Cientifica de Estudiantes de Medicina, Universidad Nacional de Trujillo, Trujillo 13011, Peru; 4 Grupo Peruano de Investigación Epidemiológica, Unidad para la Generación y Síntesis de Evidencias en Salud, Universidad San Ignacio de Loyola, Lima 15012, Peru; 5 Centro de Excelencia en Investigaciones Económicas y Sociales en Salud, Universidad San Ignacio de Loyola, Lima 15012, Peru; 6 Research Unit, Universidad Continental, Huancayo 12000, Peru; 7 Grupo de Investigación Biomedicina, Faculty of Medicine, Fundacion Universitaria Autonoma de las Americas, Pereira 660003, Risaralda, Colombia; 8 Institucion Universitaria Vision de las Americas, Pereira 660003, Risaralda, Colombia; 9 Master of Clinical Epidemiology and Biostatistics, Universidad Cientifica del Sur, Lima 15024, Peru

**Keywords:** monkeypox, vaccination acceptance, public health, systematic review

## Abstract

Vaccination it is considered a vital strategy in order to mitigate monkeypox by protecting from severe disease and helping in reduction of hospitalisations. In this sense, this study aims to estimate the global prevalence of vaccination acceptance against monkeypox. We conducted a systematic review with a comprehensive search strategy for the following databases: PubMed, Scopus and Web of Science. A random-effect model meta-analysis was carried out using observational studies assessing the intention of vaccines against monkeypox from multiple continents. The quality assessment was developed using the Newcastle-Ottawa Scale adapted for cross-sectional studies. In addition, a subgroup analysis by study location and population and a sensitivity analysis was developed.Eleven cross-sectional studies were included. A total of 8045 participants were included. The pooled prevalence of monkeypox vaccination acceptance in all participants was 56.0% (95%CI: 42.0–70.0%). In the subgroup analysis of monkeypox vaccine acceptance according to continents, the prevalence of vaccine acceptance was 50.0% (95%CI: 24.0–76.0%) in Asian countries and 70.0% (95%CI: 55.0–84.0%) in European countries. The prevalence of vaccine acceptance was 43.0% (95%CI: 35.0–50.0%) in the general population, 63.0% (95%CI: 42.0–70.0%) in healthcare workers, and 84.0% (95%CI: 83.0–86.0%) in the LGBTI community. Despite the high prevalence of monkeypox vaccination acceptance in the LGBTI community found in our study, vaccination acceptance from healthcare workers and the general population are lower. Governments could use these results for planning, developing or promoting vaccination strategies and public health policies focused on these populations.

## 1. Introduction

Monkeypox is considered a viral re-emerging zoonotic disease. This threat was declared a Public Health Emergency of International Concern, after meetings of the Emergency Committee under the International Health Regulations, by the World Health Organization, on 23 July 2022 [1,2]. Up to 25 October 2022, there have been reported 75,885 confirmed cases, 74,994 in more than 101 countries not previously reporting monkeypox [3,4,5,6]. Although in most cases, the disease is self-limited [6,7], some patients may evolve due to risk factors to complicated forms and severe illness that may lead to fatal outcomes [3,8]. In 2022, a total of 34 deaths, 21 in countries not considered endemic for monkeypox, have been reported. Therefore, in addition to enhanced case definition, clinical, epidemiological, molecular and genomic surveillance, education, and prevention are critical for disease control [9,10]. That also includes the use of vaccines against monkeypox in specific populations (e.g., LGBTI community and health care workers) under certain circumstances and approaches (e.g., ring vaccination) [11,12].

Globally, efforts to develop effective and safe vaccines are ongoing, with the already use of licensed vaccines being used in countries such as the United States, Canada, and the United Kingdom, among others, for high-risk populations. However, as occurred with SARS-CoV-2/COVID-19, to reach the appropriate vaccination coverage, the willingness to receive the vaccine from people is an essential aspect that vaccination programs should consider [13,14,15].

Even though vaccines are essential for controlling communicable diseases such as monkeypox [16,17], there are multiple motivations for reluctance to vaccines, including fear to their negative adverse effects, misinformation and its impact, or distrust of medical personnel or the health system, among others [18,19]. Likewise, sociodemographic factors such as age, sex, and geographical residence zone would be connected differently with vaccine disposition subject on the context where them are investigated [20,21]. During the multi-country outbreak, monkeypox surveys have identified vaccine-hesitant subgroups [22,23,24,25].

Vaccine hesitancy is not a new public health issue and predates the COVID-19 pandemic but also other emerging and re-emerging disease outbreaks, including monkeypox [26,27]. Thus, the current systematic review aims to estimate the prevalence of the vaccination intention for monkeypox. This information could target interventions to promote vaccination, especially among risk groups.

## 2. Methods

### 2.1. Registration and Reporting

A short version of the protocol of this systematic review was uploaded to the International Prospective Register of Systematic Reviews (PROSPERO) [CRD42022363406]. The manuscript followed the Preferred Reporting Items for Systematic Reviews and Meta-Analyses (PRISMA) statement for reporting the results [28].

### 2.2. Search Strategy and Databases

The search strategy was built using MeSH, Emtree, free terms and following the Peer Review of Electronic Search Strategies (PRESS) Checklist [29]. Afterwards, it was adapted for all the databases employed, and no date or language restrictions were applied. In addition, we developed a hand-search in preprints databases and the list of references of the included studies. Finally, the systematic search was performed on 20 September 2022, in the following databases: PubMed, Scopus, Embase, Ovid Medline, and Web of Science. The complete search strategy is attached as Supplemental Material (Table S1).

### 2.3. Study Selection and Data Extraction

At least two authors independently screened all study selection and critical appraisal phases. The eligibility criteria included (i) cross-sectional studies assessing the (ii) prevalence of vaccination intention against monkeypox. We excluded narrative reviews, scoping reviews, systematic reviews, and conference abstracts. Duplicates were removed with EndNote 20.1© (Clarivate, London, UK) and Rayyan QCRI (Rayyan Systems Inc.©, Cambridge, MA, USA) [30]. The remaining references were exported to Rayyan for screening by titles and abstracts. After identifying the potential references to be included, two authors (J.R.U.-B. and A.A.-C.) independently assessed the full text of each one. Any conflict or discrepancy in any phase of the study selection process was resolved by consensus. Data extraction was independently performed by two authors (E.A.H-B. and E.A.A.B.) using a standardised data extraction sheet designed in Google Sheets© (Google, Mountain View, CA, USA). The following information was extracted: author, publication date, country, type of population, sample size, survey modality, the prevalence of vaccination intention, and prevalence of vaccination hesitancy.

### 2.4. Risk of Bias and Publication Bias

Two authors performed the quality assessment process independently (J.R.U.-B. and E.A.H.-B.). We used the adapted version of the Newcastle-Ottawa Scale for cross-sectional studies (NOS-CS). A score ≥7 stars were considered a low risk of bias, while a score <7 stars was considered a high risk of bias. The publication bias assessment was not performed as it is not recommended in proportional meta-analysis for the following reasons: (i) conventional funnel plots and Egger’s test are inaccurate for these analyses, and (ii) there is no evidence that proportions adjust correctly to funnel plots or Egger’s tests [31,32].

### 2.5. Data Synthesis

The statistical analysis was performed using STATA 17.0 © (StataCorp LLC, College Station, TX, USA). We conducted a pooled analysis of the prevalence of vaccination intention and vaccination hesitancy against monkeypox. The 95% Confidence Intervals (CI) for the proportions reported in each study were calculated using the Clopper-Pearson Method. The Freeman-Tukey Double Arcsine Transformation was used as the variance stabiliser. We used a random-effects model (Dersimonian and Laird) for the quantitative analysis. We assessed the between-study heterogeneity using Cochran’s *Q* test and the I^2^ statistic. Values equal to or greater than 60% were defined as high heterogeneity for the I^2^ statistic, and *p*-values < 0.05 were considered indicators of heterogeneity in Cochran’s *Q* test. In addition, we carried out subgroup analysis by continents and population groups and a sensitivity analysis excluding those studies with a high risk of bias.

## 3. Results

### 3.1. Study Selection

**Figure 1:**
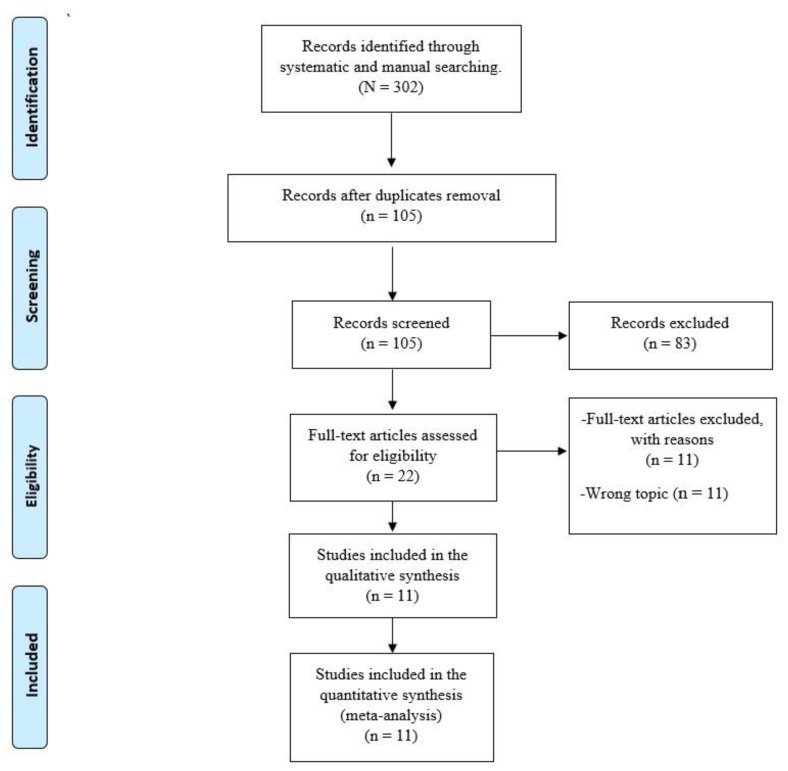
PRISMA Flow Diagram.

A total of 302 articles were identified through systematic and manual searches. Of these, 105 articles remained after eliminating 197 duplicate records. Finally, a careful assessment of the complete texts found 11 studies that fully complied with the eligibility criteria [33,34,35,36,37,38,39,40,41,42,43]. The detailed flow chart of the literature selection is shown in Figure 1.

### 3.2. Study Characteristics

The characteristics of the included studies are summarized in Table 1. All the included studies were of cross-sectional design. A total of 8045 participants were included, of whom 4855 were men and 3300 were women. The studies were published between 2020 and 2022. Two studies were carried out in Indonesia, two in Saudi Arabia, one in Iraq, one in the United States, one in the Netherlands, one in the United Kingdom, one in Nigeria, one in Italy, and one in France and Belgium. The age of all participants ranged from 18 to 57 years. All the questionnaires were administered through an online survey and developed in different populations, such as the general population, healthcare workers, and the LGBTI community.

The NOS was used for the quality assessment of the studies (see Supplementary Material Table S2). Two studies were at high risk of bias, and nine were at low risk.

### 3.3. Prevalence of Monkeypox Vaccination Acceptance

**Figure 2:**
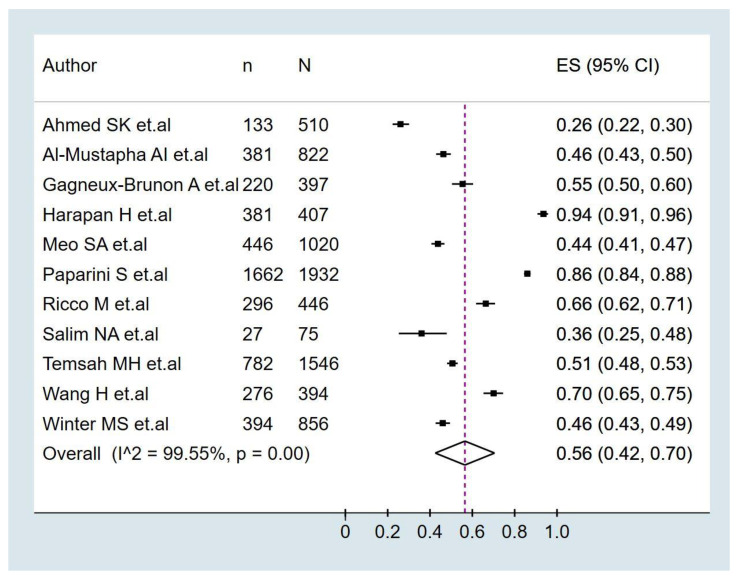
Prevalence of monkeypox vaccination intention in all participants [33,34,35,36,37,38,39,40,41,42,43].

**Figure 3:**
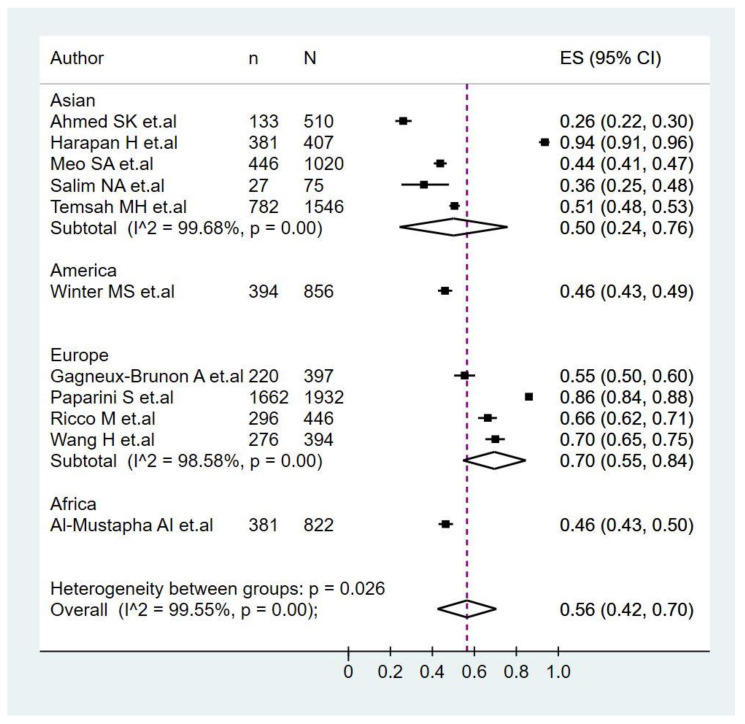
Subgroup analysis of monkeypox vaccine acceptance according to continents [33,34,35,36,37,38,39,40,41,42,43].

**Figure 4:**
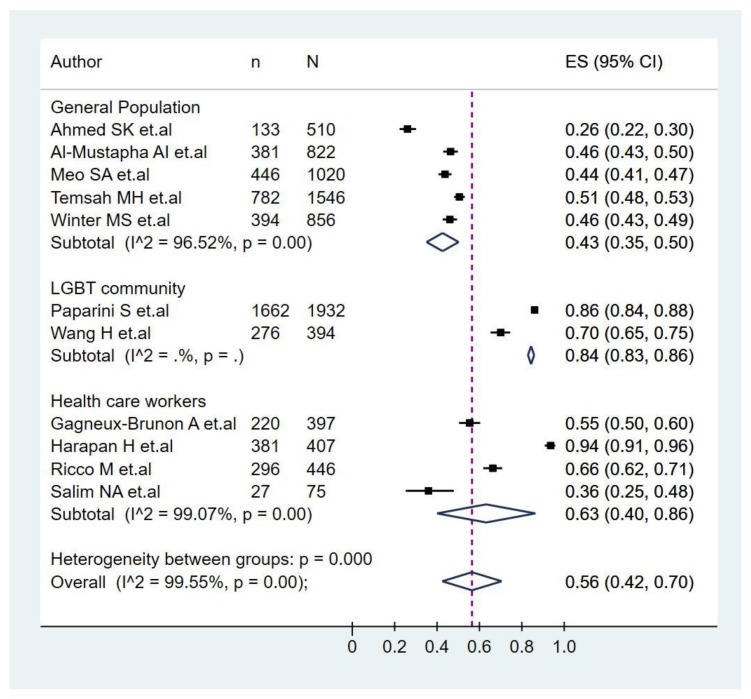
Analysis of monkeypox vaccine acceptance subgroups according to the target population of the studies [33,34,35,36,37,38,39,40,41,42,43].

The pooled prevalence of monkeypox vaccination acceptance in all participants was 56.0% (95%CI: 42.0–70.0%), with significant heterogeneity among studies (I^2^ = 99.5%) (Figure 2). In the subgroup analysis of monkeypox vaccine acceptance according to continents (Figure 3), the prevalence of vaccine acceptance was 50.0% (95%CI: 24.0–76.0%) in Asian countries and 70.0% (95%CI: 55.0–84.0%) in European countries. Meanwhile, in the subgroup analysis of monkeypox vaccine acceptance according to the target population of the studies (Figure 4), the prevalence of vaccine acceptance was 43.0% (95%CI: 35.0–50.0%) in the general population, 63.0% (95%CI: 42.0–70.0%) in healthcare workers, and 84.0% (95%CI: 83.0–86.0%) in the LGBTI community. In the sensitivity analysis, after removing studies with a high risk of bias, the prevalence of vaccine acceptance was 60.0% (95%CI: 44.0–75.0%), with no decrease in heterogeneity (I^2^ = 99.61%).

### 3.4. Prevalence of Monkeypox Vaccination Hesitancy

**Figure 5:**
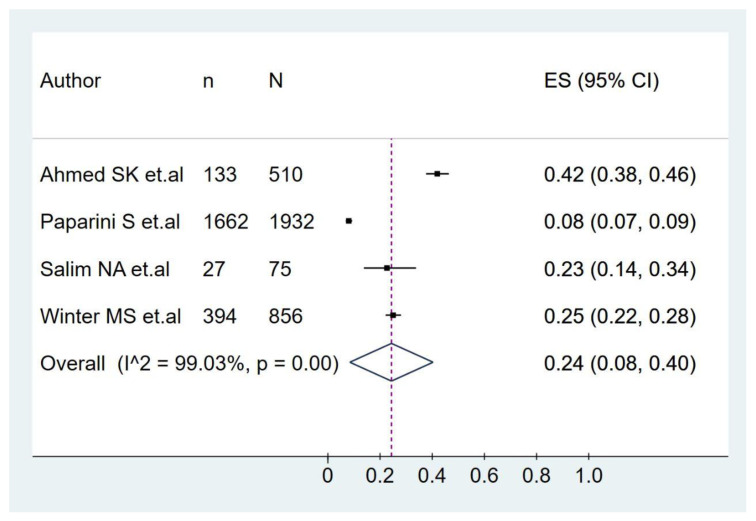
Prevalence of monkeypox vaccination hesitancy [33,34,40,43].

The pooled prevalence of monkeypox vaccination hesitancy in all participants was 24.0% (95%CI: 8.0–40.0%), with significant heterogeneity among studies (I^2^ = 99.03%) (Figure 5).

## 4. Discussion

Better vaccination coverages are in general important for most diseases where vaccines are currently available. Then, it is not only important to develop efficacious and safe vaccines, but also to warrant the appropriate logistical issues, fair distribution and the populations acceptance in order to have the necessary demand of them [44]. Then vaccine hesitance and acceptance is a key determinant of vaccination coverage that should be assessed and consequently addressed with evidence, education and promotion as part of disease prevention campaigns, included now for monkeypox.

Our systematic review targeted to estimate the prevalence of monkeypox vaccination acceptance globally. Present findings indicated a moderate prevalence of monkeypox vaccine acceptance (56%), which was, as expected, higher in Europe (70%) and lower in Asia (50%) due to the incidence and probably associated perception of risk and impact. Nations such as Spain, the United Kingdom, France, and Germany are located in the top ten of incidence. Indeed, in May 2022, the outbreak started in the United Kingdom, and multiple European countries were rapidly affected. Still, multiples countries in Asia have not reported cases of monkeypox, and among those with confirmed patients, numbers would be low (e.g., India with 17; Thailand, 11; Saudi Arabia, 8; Japan, 7; Qatar, 5; China, 1) [24,45]. Although among the included studies in the present systematic reviews, there were studies from Asia, Europe, the USA, and Africa, but not Latin America. Before 2022, only one study addressed the acceptance of monkeypox vaccines. That is consistent with the past lack of studies on monkeypox and that monkeypox has been neglected [46,47,48,49].

A number of local, racial, religious, cultural and a number of other aspects may influence people’s perception on their acceptance of vaccination, as well as misinformation, as was clearly observed in the COVID-19 pandemic [50,51,52,53,54,55,56,57].

Also, as expected, the acceptance of monkeypox vaccines was low in the general population (43%), even may perceive this disease as only occurring in the LGBTI community, with its associated stigma [58]. However, at the same time, it was higher among the LGBTI population (84%), which has been predominantly affected by monkeypox [59,60,61]. LGBTI communities should be prioritized for education and promotion on monkeypox, its transmission and preventive practices, while their stigma should be avoided [62,63].

Curiously, although the general belief is that the acceptance should be 100% among healthcare workers, especially physicians, this systematic review found only 64%. This was even lower than studies for COVID-19 vaccine acceptance among physicians in different parts of the world, e.g., Colombia (90.7%) [64], and comparable with results from India (63.34%) [65].

The overall acceptance of monkeypox vaccines (56%) was lower than in some systematic reviews for COVID-19. For example, a systematic review, including 19 studies from Latin America, found that accepting COVID-19 vaccines was 78% [13].

A recent study on acceptance for the monkeypox vaccines among 32,902 male owners of smartphones, registered at online gay-dating apps in Europe found that the acceptance was 82% and higher in north (84.8–90.4%) and western (83.1–87.7%) countries, compared to south-east (60.9–70.2%) and eastern (59.9–71.1%) European countries [66].

All these findings underscore the relevance of education and the promotion of vaccines. The efficacy and safety of available vaccines against monkeypox should be divulgated among the general population, but especially among healthcare workers and LGBTI communities, which are risk groups. As the attitudes towards different vaccines as well as the trust degree and misinformation are probable to change through different periods according to outcomes and opinions, they require to be constantly assessed, considering their evolving nature. Challenges and attitudes towards vaccines will evolve and require close monitoring to improve compliance and distribution [19]. As far as the outbreak evolves, effectiveness studies are urgently required to improve the understanding of the real impact of the monkeypox vaccines. In the meantime, a recent study in the Netherlands found that among those non-primed vaccinated individuals, with a 2-shot immunization series with the modified vaccinia virus Ankara-Bavarian Nordic (MVA-BN, also known as Jynneos, Imvanex or Imvamune), this estimulated partially low titers of MPXV-protective neutralizing antibodies. Also, that the MVA-based influenza vaccine dose-sparing leads to low levels of MPXV-neutralizing antibodies, whereas a third dose with the same vaccine significantly enhance the antibody immune response. As the importance of MPXV-neutralizing antibodies as a possible protection correlate for disease and transmission is yet to be defined accurately, based on that preliminary data, authors concluded that follow-up studies at cohorts of vaccinated subjects would be important to evaluate vaccine protection in populations at risk [67].

Globally, nations, particularly those more affected by the monkeypox outbreak, must prioritise implementing strategies to deal with misinformation and vaccination hesitancy [26]. Current findings are key for the implementation of vaccination policies. Providing evidence-based data in means and forms understandable to everybody is key; regarding this, public health authorities and professionals should increase their use of social media, as these are value resources yet to be a more significant tool in disease prevention. Therefore, national and regional authorities should enhance their efforts in providing massively intense educational interventions, including higher presence in social and news media that are also key in providing trustworthy information to the population that needs to be vaccinated promptly and reduce the risk of monkeypox, especially in risk groups.

As occurred with COVID-19 [13], identifying specific groups with a lower rates of vaccination intention for monkeypox would help health authorities and governments to explore and design more efficient public health approaches for vaccination. Our study results constitute an input towards the implemented measures for global vaccination against monkeypox. They could be used as a guide for developing new public health policies focused on population subgroups with a low prevalence of monkeypox vaccination acceptance. Additionally, these results could be used as a reference in future outbreaks to stratify groups with low vaccine acceptance and build specific strategies for them. Further research is needed to study factors associated with low monkeypox vaccination acceptance among these groups.

Our systematic review has limitations, the main one being the high heterogeneity in the meta-analyses performed, which would not explained using the subgroup and sensitivity analyses. The high heterogeneity probably has to do with the differences in the populations surveyed as well as with the measurement instruments used. Likewise, we found few studies in America and Africa, so it is necessary to carry out more research in these continents.

## 5. Conclusions

Despite the high rate of monkeypox vaccination acceptance in the LGBTI community found in our study, the prevalence of vaccination from healthcare workers and the general population is lower. Governments could use these results for designing, developing or promoting vaccination approaches and public health policies focused on these vulnerable and at-risk populations. Immunoprevention remains an essential public health intervention to prevent disease and probably transmission, even in monkeypox.

## Figures and Tables

**Table 1:** Characteristics of the included studies.

Author	Year of Publication	Country	Target Population	Participants (Male/Female/Nonbinary)	Age (Mean ± SD or Age Ranges)	Survey Type	Data Collection Date	Response Recorded as Vaccine Acceptance	Prevalence of Vaccination Acceptance	Prevalence of Monkeypox Vaccination Hesitancy
Ahmed SK, et al. [33]	2022	Iraq	General population	510 (277/233)	18–57	Online survey	27 July 2022–30 July 2022	Yes	26%	42%
Winter MS, et al. [34]	2022	United States	General population	856 (419/437)	NR	Online survey	June 2022	Yes	46%	25%
Wang H, et al. [35]	2022	Netherlands	LGBTI community	394 (394/0)	NR	Online survey	July 2022	High/very high vaccination intention (scale 4 and 5)	70%	NR
Gagneux-Brunon A, et al. [36]	2022	France and Belgium	Healthcare workers	397 (101/296)	43.3(12)	Online survey	15 June 2022–8 August 2022	Intention to get vaccinated	55.4%	NR
Salim NA, et al. [43]	2022	Indonesia	Healthcare workers	75 (49/26)	26–38	Online survey	2 August 2022–5 August 2022	Yes	36%	22.70%
Ricco M, et al. [37]	2022	Italy	Healthcare workers	446 (149)	42.9 (10)	Online survey	4 May 2022–31 May 2022	Totally Agree/Agree	66.36%	NR
Meo SA et al. [38]	2022	Saudi Arabia	General population	1020 (466/554)	NR	Online survey	15 May 2022–15 July 2022	Yes	43.7%	NR
Temsah MH, et al. [39]	2022	Saudi Arabia	General population	1546 (650/896)	NR	Online survey	27 May 2022–5 June 2022	Yes	50.6%	NR
Paparini S, et al. [40]	2022	United Kingdom	LGBTI community	1932 (1750/88/94)	NR	Online survey	15 June 2022–27 July 2022	Yes	86%	8%
Harapan H, et al. [41]	2020	Indonesia	Healthcare workers	407 (128/279)	NR	Online survey	25 May 2019–25 July 2019	Yes	93.6%	NR
Al-Mustapha AI, et al. [42]	2022	Nigeria	General population	822 (472/342/8)	NR	Online survey	16 August 2022–29 August 2022	NR	46.37%	NR

NR: Not Reported.

## Data Availability

Available on reasonable request.

## Supplementary Materials

The following supporting information can be downloaded at: https://www.mdpi.com/article/10.3390/pathogens11111248/s1, Table S1: Search strategies; Table S2: Quality assessment of included studies; Figure S1. Sensitivity analysis of monkeypox vaccine acceptance according to the risk of bias.
