# Supplementary material for: Acceptance towards Monkeypox Vaccination: A Systematic Review and Meta-Analysis
Source: Pathogens. 2022 Oct 28;11(11):1248. doi: 10.3390/pathogens11111248 (PMC9697127; doi:10.3390/pathogens11111248)
Supplement: Supplementary file 1 [file pathogens-11-01248-s001.zip › pathogens-2001098-supplementry.pdf]

Table S1. Search strategies

Source	PubMed
**Search**	**Formula**
#1	Monkeypox virus [MH] OR Monkeypox [MH] OR “monkeypox*” [TIAB] OR (“monkey*” [TIAB] AND “pox*” [TIAB]) OR “chimpanzeepox*” [TIAB]
#2	Vaccination [MH] OR vaccin* [TIAB] OR immunization* [TIAB] OR immunisation* [TIAB]
#3	#1 AND #2
**Source**	**Scopus**
**Search**	**Formula**
#1	TITLE-ABS-KEY (“monkeypox*” OR (“monkey*” W/3 “pox*”) OR “chimpanzeepox*”)
#2	TITLE-ABS-KEY(“vaccin*” OR “immunization*” OR “immunisation*”)
#3	#1 AND #2
**Source**	**Web of Science**
**Search**	**Formula**
#1	TI=(“monkeypox*” OR (“monkey*” NEAR/3 “pox*”) OR “chimpanzeepox*”) OR AB=(“monkeypox*” OR (“monkey*” NEAR/3 “pox*”) OR “chimpanzeepox*”) OR AK=(“monkeypox*” OR (“monkey*” NEAR/3 “pox*”) OR “chimpanzeepox*”) OR KP=(“monkeypox*” OR (“monkey*” NEAR/3 “pox*”) OR “chimpanzeepox*”) OR TS=(“monkeypox*” OR (“monkey*” NEAR/3 “pox*”) OR “chimpanzeepox*”)
#2	TI=(“vaccin*” OR “immunization*” OR “immunisation*”) OR AB=(“vaccin*” OR “immunization*” OR “immunisation*”) OR AK=(“vaccin*” OR “immunization*” OR “immunisation*”) OR KP=(“vaccin*” OR “immunization*” OR “immunisation*”) OR TS=(“vaccin*” OR “immunization*” OR “immunisation*”)
#3	#1 AND #2
**Source**	**Embase**
**Search**	**Formula**
#1	'monkeypox'/exp OR (“monkeypox*” OR (“monkey*” NEAR/3 “pox*”) OR “chimpanzeepox*”):ti OR (“monkeypox*” OR (“monkey*” NEAR/3 “pox*”) OR “chimpanzeepox*”):ab OR (“monkeypox*” OR (“monkey*” NEAR/3 “pox*”) OR “chimpanzeepox*”):kw
#2	‘vaccination’/exp OR (‘vaccin*’ OR ‘immunization*’ OR ‘immunisation*’):ti OR (‘vaccin*’ OR ‘immunization*’ OR ‘immunisation*’):ab OR (‘vaccin*’ OR ‘immunization*’ OR ‘immunisation*’):kw
#3	#1 AND #2
**Source**	**OVID**
**Search**	**Formula**
#1	(monkeypox* OR (monkey* adj3 pox*) OR chimpanzeepox*).ti. OR (monkeypox* OR (monkey* adj3 pox*) OR chimpanzeepox*).ab. OR (monkeypox* OR (monkey* adj3 pox*) OR chimpanzeepox*).kw.
#2	(vaccin* OR immunization* OR immunisation*).ti. OR (vaccin* OR immunization* OR immunisation*).ab. OR (vaccin* OR immunization* OR immunisation*).kw.
#3	#1 AND #2

Table S2. Quality assessment of included studies.

NEWCASTLE - OTTAWA QUALITY ASSESSMENT SCALE FOR CROSS-SECTIONAL STUDIES									
STUDY	**SELECTION**				**COMPARABILITY**	**OUTCOME**			
	**Representativeness of the sample**	**Sample size**	**Non-respondents**	**Ascertainment of the exposure (risk factor)**	**The subjects in different outcome groups are comparable, based on the study design or analysis. Confounding factors are controlled. Maximum : ☆☆**	**Assessment of outcome Maximum : ☆☆**	**Statistical test**	**SCORE**	**Evidence quality**
Ahmed SK et.al [33]	☆	☆	☆	☆	☆☆	☆	☆	*8*	Low risk of bias
Winter MS et.al[34]	☆	☆		☆	☆	☆	☆	*6*	High risk of bias
Wang H et.al[35]	☆	☆	☆	☆	☆☆	☆	☆	*8*	Low risk of bias
Gagneux-Brunon A et.al[36]	☆	☆	☆	☆	☆	☆	☆	*7*	Low risk of bias
Salim NA et.al[43]	☆	☆		☆	☆	☆	☆	*6*	High risk of bias
Ricco M et.al[37]	☆	☆	☆	☆	☆☆	☆	☆	*8*	Low risk of bias
Meo SA et.al[38]	☆	☆	☆	☆	☆☆	☆	☆	*8*	Low risk of bias
Temsah MH et.al[39]	☆	☆	☆	☆	☆☆	☆	☆	*8*	Low risk of bias
Paparini S et.al[40]	☆	☆	☆	☆	☆	☆	☆	*7*	Low risk of bias
Harapan H et.al[41]	☆	☆	☆	☆	☆	☆	☆	*7*	Low risk of bias
Al-Mustapha AI et.al[42]	☆	☆	☆	☆	☆	☆	☆	*7*	Low risk of bias

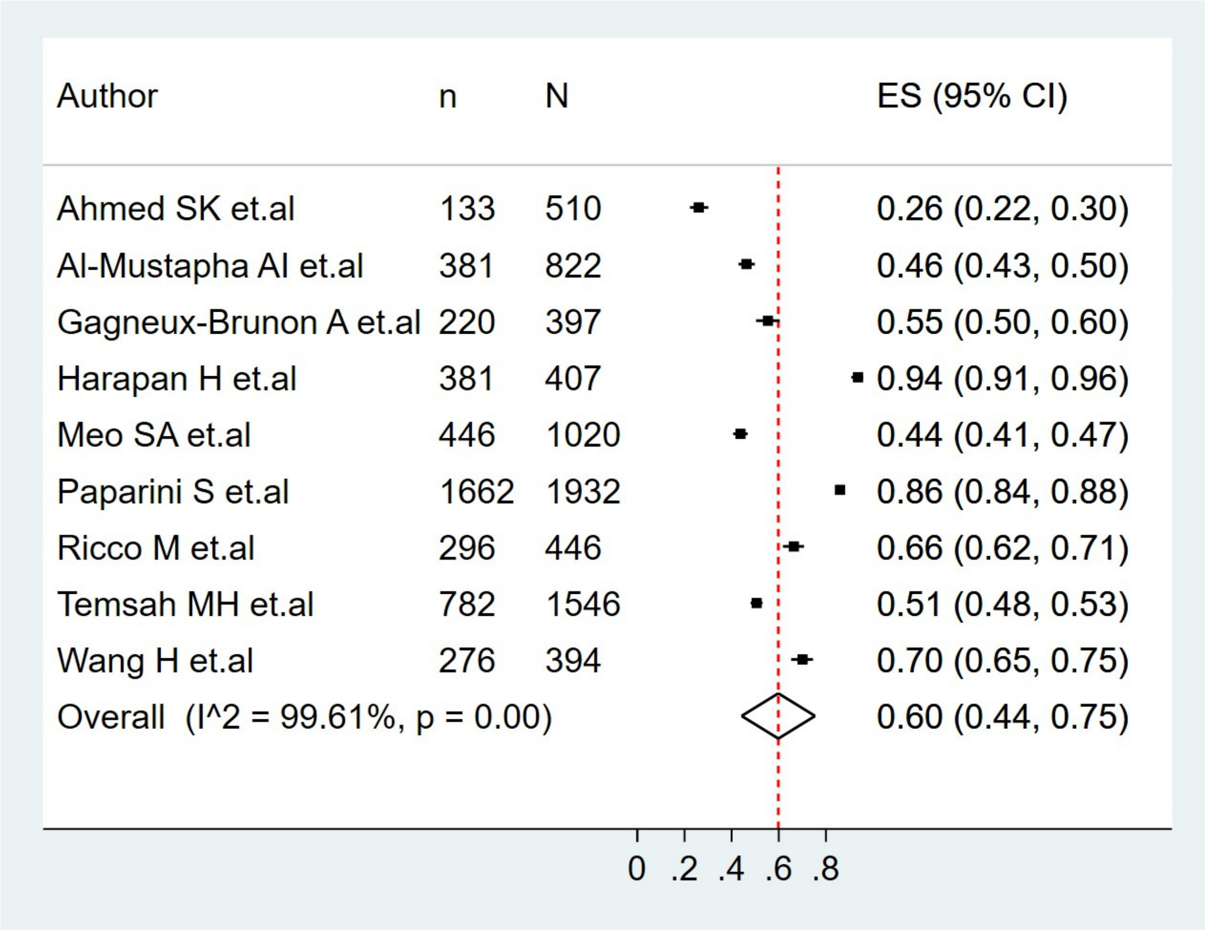

Figure S1. Sensitivity analysis of monkeypox vaccine acceptance according to the risk of bias.[33,35–42]
